# Metal organic framework derived NaCo_x_O_y_ for room temperature hydrogen sulfide removal

**DOI:** 10.1038/s41598-021-94265-7

**Published:** 2021-07-19

**Authors:** Nishesh Kumar Gupta, Jiyeol Bae, Kwang Soo Kim

**Affiliations:** 1 University of Science and Technology (UST), Daejeon, Republic of Korea; 2 Department of Land, Water, and Environment Research, Korea Institute of Civil Engineering and Building Technology (KICT), Goyang, Republic of Korea

**Keywords:** Pollution remediation, Inorganic chemistry

## Abstract

Novel NaCo_x_O_y_ adsorbents were fabricated by air calcination of (Na,Co)-organic frameworks at 700 °C. The NaCo_x_O_y_ crystallized as hexagonal microsheets of 100–200 nm thickness with the presence of some polyhedral nanocrystals. The surface area was in the range of 1.15–1.90 m^2^ g^−1^. X-ray photoelectron spectroscopy (XPS) analysis confirmed Co^2+^ and Co^3+^ sites in MOFs, which were preserved in NaCo_x_O_y_. The synthesized adsorbents were studied for room-temperature H_2_S removal in both dry and moist conditions. NaCo_x_O_y_ adsorbents were found ~ 80 times better than the MOF precursors. The maximum adsorption capacity of 168.2 mg g^−1^ was recorded for a 500 ppm H_2_S concentration flowing at a rate of 0.1 L min^−1^. The adsorption capacity decreased in the moist condition due to the competitive nature of water molecules for the H_2_S-binding sites. The PXRD analysis predicted Co_3_S_4_, CoSO_4_, Co_3_O_4_, and Co(OH)_2_ in the H_2_S-exposed sample. The XPS analysis confirmed the formation of sulfide, sulfur, and sulfate as the products of H_2_S oxidation at room temperature. The work reported here is the first study on the use of NaCo_x_O_y_ type materials for H_2_S remediation.

## Introduction

Hydrogen sulfide (H_2_S) is a toxic malodorous gas originating from different natural and anthropogenic activities, including volcanic eruption, microbial degradation of vegetation, natural gas and oil industries, and sewage treatment facilities. H_2_S creates human discomfort even at a low concentration of 0.1 ppm. Long-term exposure to 50 ppm causes respiratory tract irritation, whereas a range of 500–1000 ppm could lead to death^1, 2^. H_2_S presence in various energy sources like natural gas, petroleum oil, and biogas is the major concern for industries as H_2_S corrodes transport pipelines and poison catalysts^3^. The combustion of H_2_S-rich fuel releases sulfur dioxide in the atmosphere, which eventually leads to the formation of aerosol sulfates and sulfuric acid by reacting with air components^4^. The presence of aerosol sulfates and sulfuric acid in the atmosphere adversely affects human health and the environment by acidifying oceans, lakes, and soil. Thus, mitigation strategies are required to remove H_2_S from energy gases.

Metal–organic frameworks (MOFs) are inorganic–organic hybrid materials formed by the self-assembly of metal ions and bridging organic ligands. Unique physicochemical properties like large surface area and pore volume, good structural stability, and ease of fabrication have popularized their use in various environmental applications^5, 6^. MOFs are being used as precursors for the fabrication of porous metal oxides with application-specific structural and functional characteristics^7–11^. MOFs transformation to metal oxides has provided an alternative pathway to develop metal oxides with newer morphologies, high surface area, and high porosity^12^.

Unlike oxides of Fe, Cu, and Zn, oxides and hydroxides of cobalt are less explored for room temperature desulfurization. Xue et al. screened several transition metal oxides for room temperature H_2_S removal. The study reported 6 mg g^−1^ of H_2_S uptake capacity for Co_3_O_4_, which increased to 134 mg g^−1^ for Zn_3.5_CoO_4.9_ mixed oxide^13^. Since the surface area of Co_3_O_4_ was undetermined, the low adsorption capacity was probably due to its low surface area. This inference was supported by the reported work of Pahalagedara et al. The study reported mesoporous Co_3_O_4_ with a surface area of 143 m^2^ g^−1^ and an adsorption capacity of 134 mg g^−1^ at room temperature^14^. Wang and coworkers integrated Co_3_O_4_ in three-dimensionally ordered macroporous silica for room temperature desulfurization process, where the adsorption capacity reached as high as 189 mg g^−1^^15^. Besides Co_3_O_4_, CoOOH^16^ and Co(OH)_2_^17^ integrated with graphite oxide has been successfully tested for the room temperature desulfurization process. Long et al. developed aerogels and xerogels of Na-MnO_x_ and H-MnO_x,_ which had the H_2_S adsorption capacity in the range of 17–680 mg g^−1^^18^. Also. Some reports are available for the application of cobalt-based MOFs like ZIF-67 in the desulfurization process^19^. Though Co-MOFs and MOF-derived cobalt oxides have not been studied for H_2_S adsorptive/oxidative removal, Dong et al. have reported cataluminescence sensing of H_2_S using ZIF-67-derived porous Co_3_O_4_ dodecahedra.

The product of MOF pyrolysis/calcination depends on the temperature, metal ions, substrates, and rate of heating^20, 21^. While the calcination of monometallic MOFs yields single-phase oxides, the presence of a second metal in the MOF has unpredictable outcomes. Huang et al. reported the formation of CuCr_2_O_4_/CuO composite after the calcination of bimetallic Cr-embedded MOF-199 at 600 °C^9^. Yang et al. reported the formation of CoFe_2_O_4_ nanocrystals after the air calcination of bimetallic Co-Fe terephthalate MOF at 400 °C^22^. Lee and Kwak reported the formation of Mn-doped Fe_2_O_3_ after air calcination of the bimetallic FeMn-MOF^21^. Thus, the formation of metal oxides as composites, single-phase, or doped oxides is highly unpredictable.

In the literature, the role of sodium in MOFs is largely unexplored, with some reports on sodium metal–organic frameworks^23^. In the present study, the unexpected presence of sodium in Co-based MOFs has played a decisive role in the formation of MOF-derived oxides. The presence of Na in Co-MOF resulted in the formation of single-phase NaCo_x_O_y_ as opposed to Co_3_O_4_/Na_2_O as the air calcination product. Apart from unique microsheets like morphology, the material showed exceptionally high H_2_S removal capacity in ambient conditions. The parameters associated with the column studies were optimized. Moreover, the H_2_S removal mechanism was studied in detail using different microscopic and spectroscopic analyses.

## Materials and methods

### Chemicals

Benzene-1,4-dicarboxylic acid (H_2_BDC), benzene-1,3,5-tricarboxylic acid (H_3_BTC), and cobalt(II) nitrate hexahydrate (Co(NO_3_)_2_·6H_2_O) were purchased from Sigma Aldrich. Ethanol, methanol, N,N-dimethyl formamide (DMF), and sodium hydroxide (NaOH) pellets were acquired from Samchun Pure Chemicals, Korea. H_2_S gas (500 ppm balanced with N_2_ gas) was procured from Union gas, Korea.

### Synthesis of adsorbents

A 4.29 g of NaOH pellets were dissolved in 82.5 mL methanol, which served as the precipitating agent. The cobalt salt solution was prepared by dissolving 12.07 g of Co(NO_3_)_2_·6H_2_O in 75 mL of DMF. The NaOH solution was added to the cobalt salt solution to form hydroxide. Then, it was kept under ultrasonication, and H_2_BDC solution (6.64 g in 190 mL of DMF) was added to it. After 20 min of ultrasonication, the CoBDC MOF product was separated, washed with ethanol, and dried at 70 °C in a hot air oven. CoBTC MOF was synthesized using the same protocol with 8.50 g of H_3_BTC in 190 mL of DMF. Finely powdered MOF in an alumina crucible was calcined in air at 700 °C for 24 h. The CoBDC and CoBTC-derived oxide was labelled as NCO-D and NCO-T, respectively.

### Material characterization

The surface morphology of materials was probed by field emission scanning electron microscopy (FE-SEM, Hitachi S-4300, Japan). A gold-platinum alloy was coated on dried samples using an E-1048 Hitachi ion sputter. The transmission electron microscopy (TEM) was conducted on a field emission TEM (FE-TEM, JEM-2010F, JEOL, Japan). Elemental mapping was done by energy-dispersive X-ray spectroscopy (EDAX, X-Maxn 80 T, Oxford, UK). N_2_ adsorption–desorption isotherms were recorded at − 196 °C over a Gemini series Micromeritics 2360 instrument and analyzed by the Brunauer–Emmett–Teller (BET) equation. Samples were pre-heated at 200 °C for 8 h for degassing purpose. XRD patterns were recorded on an X-ray diffractometer (Ultima IV Rigajku, Japan) with Cu Kα and a Ni filter. Fourier-transform infrared (FTIR) spectra were recorded on a Cary670 FTIR spectrometer after pelletization with KBr. For XPS analyses, a K-alpha XPS instrument (Thermo Scientific Inc., UK) with a monochromatic Al Kα X-ray source and 4.8 × 10^−9^ mbar of pressure was used. Spectra were charge-corrected to the main line of the carbon 1s spectrum (aromatic carbon) set to 284.7 eV. Spectra were analyzed using CasaXPS software (version 2.3.14).

### Breakthrough studies

H_2_S breakthrough studies were carried out in a fixed bed micro-reactor at 25 °C. A known mass of an adsorbent packed between glass wool was supported on silica beads in a pyrex tube (height: 50 cm, diameter: 1 cm). The adsorbents were tested in dry and moist (by passing moist air through the adsorbent bed for 0.5 h with 0.3 L min^−1^ of flowrate) conditions. The concentration of the outgoing gas was measured by a multi-gas analyzer (GSR-310, Sensoronic, Korea) every 15 s until the effluent concentration reached 10 ppm (2% was the breakthrough condition). The adsorption capacity of an adsorbent (*q*, mg g^−1^) was calculated by integration of the area above the breakthrough curve.

1 $$q = \frac{{C_{0} Q}}{m}\int_{0}^{{t_{b} }} {\left( {1 - \frac{C}{{C_{0} }}} \right)dt}$$

where *C*_0_—initial concentration (500 ppm or 0.697 mg L^−1^), *Q*—flowrate, *m*—the mass of adsorbent (g), and *t*_b_—breakthrough time.

## Results and discussion

### Characterization of adsorbents

**Figure 1:**
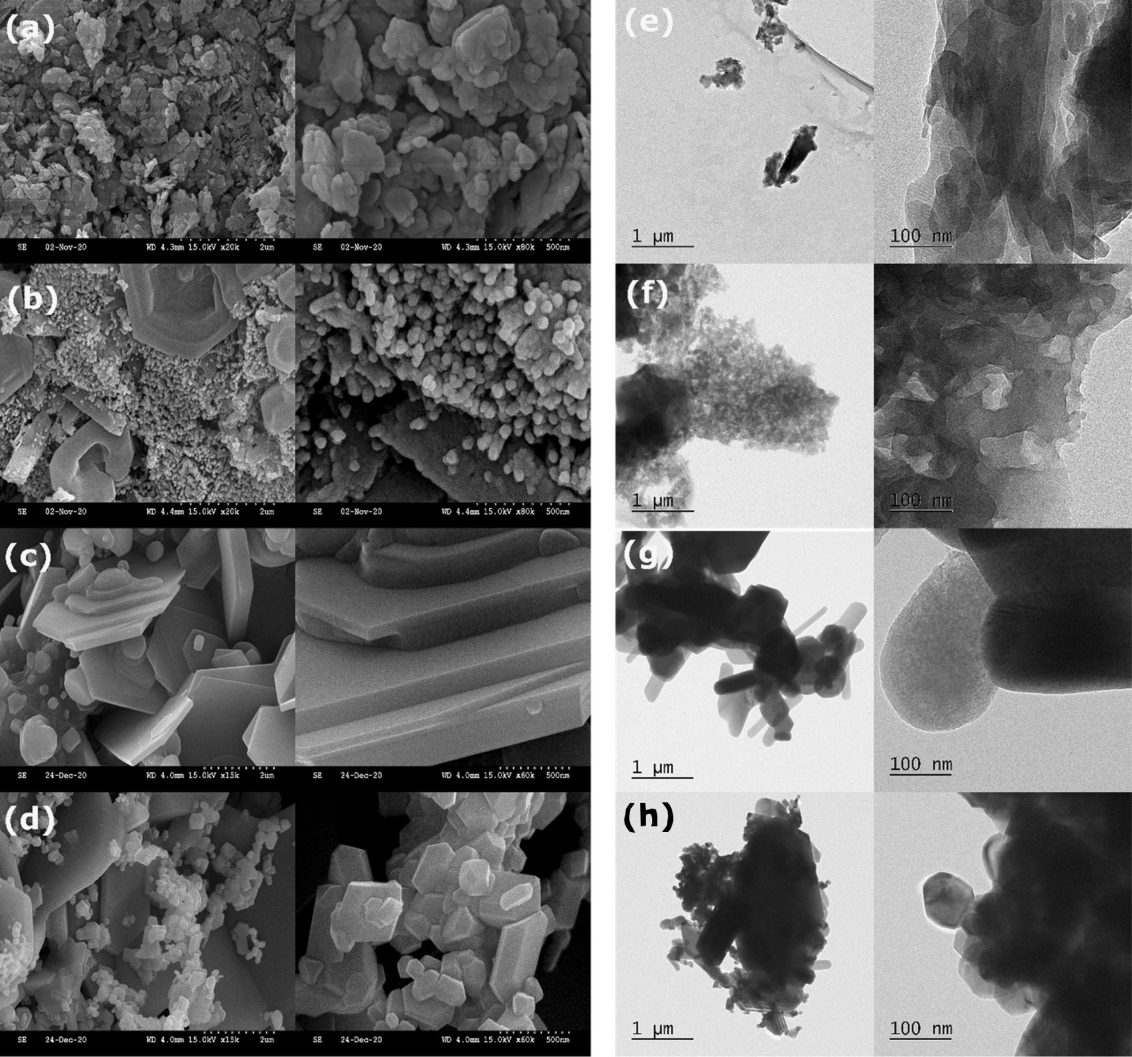
SEM images of (a) CoBDC; (**b**) CoBTC; (**c**) NCO-D; (**d**) NCO-T; TEM images of (**e**) CoBDC; (**f**) CoBTC; (**g**) NCO-D; (**h**) NCO-T.

The SEM and TEM micrographs of Co-MOFs and derived oxides are shown in Fig. 1. CoBDC has a cluttered sheet-like morphology (Fig. 1a,e). CoBTC has distorted hexagonal microcrystals surrounded by nanothreads (Fig. 1b,f). The oxides formed by the calcination of MOFs have similar morphology. The NCO-D has smooth intercalated hexagonal sheets with some deposition of nanoparticles (Fig. 1c,g). The hexagonal microsheets and nanothreads in CoBTC were transformed to irregular hexagonal microsheets and polyhedral nanoparticles, respectively, in NCO-T (Fig. 1d,h). The TEM-EDS analyses of Co-MOFs and derived oxides are shown in Figs. S1 and S2. The EDS analysis confirmed the presence of Co, C, and O with an additional Na peak at ~ 1.0 eV for Co-MOFs (Fig. S1). For CoBTC, a metal-to-ligand ratio of 1:1 (opposed to the conventional 3:2) was adopted in the present study. The deficient Co ions for metal–ligand interactions in CoBTC was balanced by Na-ligand interactions^24^. The probable consumption of some of the cobalt hydroxide to form cobalt oxide was compensated by Na ions, which interacted with the carboxylate groups in a strong alkali medium^25^. The oxides derived from the calcination of Co-MOFs (NCO-D and NCO-T) (Fig. S2) have all peaks except for carbon. Based on EDS analysis, a compositional formula of NaCo_0.7_O_2.4_ and NaCo_1.1_O_3.3_ was assigned to NCO-D and NCO-T, respectively (Table S1). The excess oxygen could be from the hydroxyl density, adsorbed molecular oxygen, and mixed-valence states of cobalt ions.

**Figure 2:**
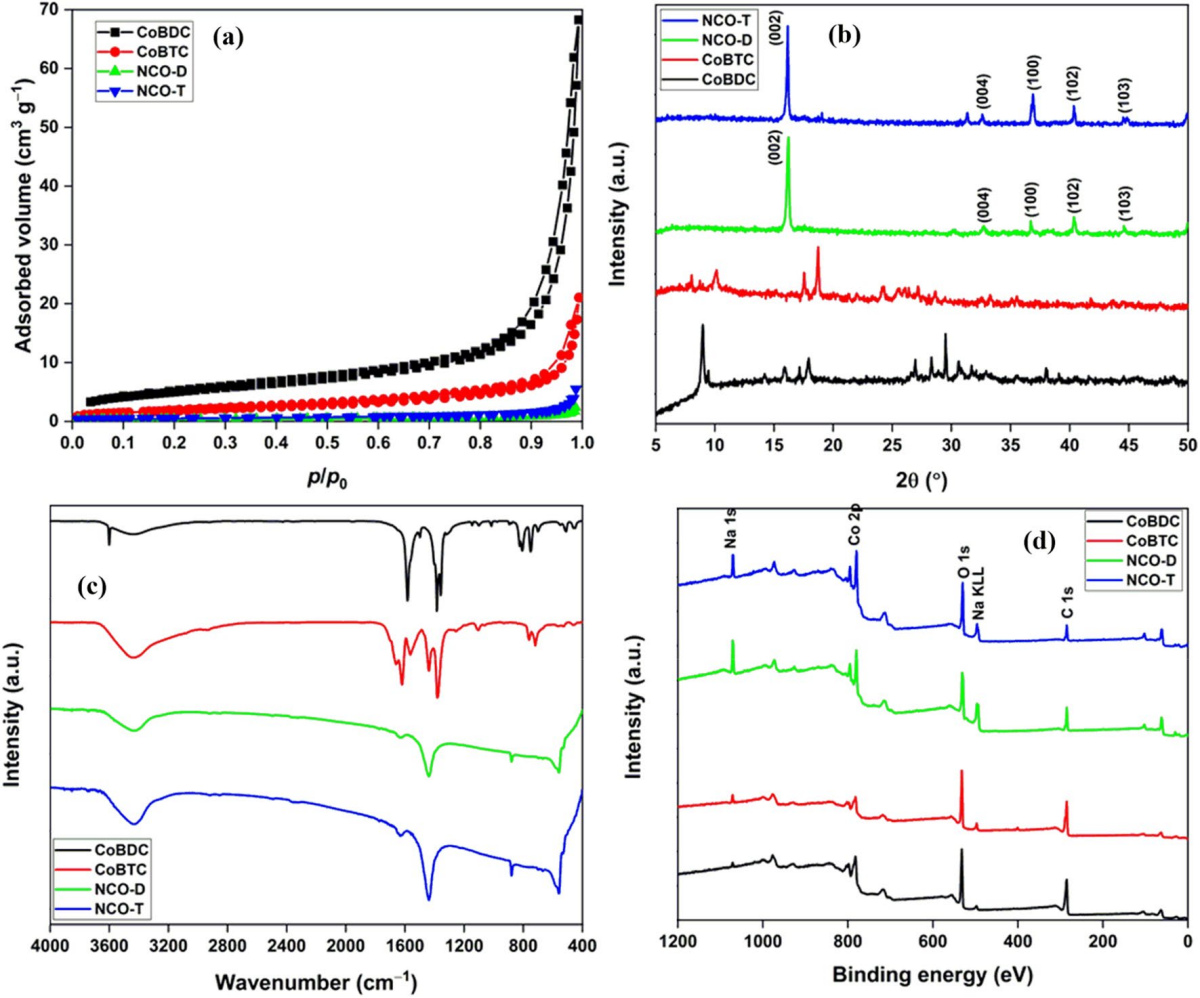
(**a**) N_2_ adsorption–desorption isotherms (**b**) PXRD patterns; (**c**) FTIR spectra; (**d**) XPS surveys of Co-MOFs and derived oxides.

The surface and pore properties of MOFs and oxides were evaluated by N_2_ adsorption–desorption isotherms (Fig. 2a). The MOFs exhibited adsorption–desorption isotherms for mesoporous materials. The surface area of these MOFs (6.9–18.3 m^2^ g^−1^) was in agreement with the reported values^25^. The low surface area of MOFs was due to the formation of microparticles^26^. The MOF-derived binary metal oxides exhibited curves for macroporous or non-porous materials^27^. Metal oxides have a lower surface area than MOFs due to complete loss of mesoporosity after high-temperature calcination. The measured surface area of NCO-D and NCO-T was 1.15 and 1.90 m^2^ g^−1^, respectively (Table S2).

The PXRD pattern of MOFs and derived oxides are shown in Fig. 2b. The PXRD pattern of CoBDC matched with the one reported by Ma et al. with a slight variation in the peak intensity at 28.5°^28^. Moreover, the peak at 9.0° has a minor split due to a slight distortion in the symmetry of MOF^29^. The PXRD pattern of CoBTC largely matched with the reported MOF by Nowacka et al. with an additional presence of diffraction peaks in the 5°–10° range^30^. Since MOFs were synthesized in a strong alkali medium, Na^+^ ions in the MOFs as the nodes had a strong impact on their PXRD pattern^31^. The calcination of these MOFs results in the formation of conventional oxides like Co_3_O_4_^32^. On the other hand, mixed metal ions in a MOF yields binary metal oxides^33^. In the present study, (Na,Co)-based MOFs oxidized to yield single-phase NaCo_x_O_y_ materials and not Co_3_O_4_/Na_2_O composite. The possible reason for single-phase NaCo_x_O_y_ formation was a high calcination temperature of 700 °C and a long heating time of 24 h. In the literature, numerous reports are available on the fabrication of sodium cobalt oxides by the solid-state synthesis method with Na_2_O_2_ and Co_3_O_4_ as precursors^34^. During the calcination of (Na,Co)-MOFs, the oxide formation occurs through the initial growth of oxide nanoparticles on the MOF surface. These nanoparticles served as seeds for the development of microsheets^35^. In the case of the delocalized distribution of Na and Co in the MOF structure, both the metal cations took part in the formation of oxide to yield NaCo_x_O_y_ type materials. On the contrary, localized distribution of Na and Co in the MOF may have formed Na_2_O/Na_2_O_2_ and Co_3_O_4_ nanoparticles, which served as the precursors for NaCo_x_O_y_ type materials at a high temperature of 700 °C for 24 h. Thus, in both cases, NaCo_x_O_y_ formation was possible. For this reason, the PXRD pattern of NaCo_x_O_y_ matched with the pattern of NaCo_2_O_4_^36^ with the absence of Na_2_O or Co_3_O_4_ (Fig. S3).

The FTIR spectra of MOFs and oxides are shown in Fig. 2c. The band at 3432 cm^−1^ was assigned to the stretching vibrations of O–H stretching vibrations of adsorbed water molecules. The high-intensity bands at 1579, 1387, and 1357 cm^−1^ were due to the asymmetric and symmetric O–C–O stretching of organic linkers. The band at 1502 cm^−1^ was for C=C stretchings of the aromatic skeleton. The mid-intensity bands at 825, 808, and 753 cm^−1^ were attributed to the C–H bending modes^37, 38^. The band at 457 and 510 cm^−1^ were due to the Co–O stretching^39^. For CoBTC, additional peak at 1668 and 1707 cm^−1^ were possibly due to the Na-bound carboxylate groups. In the FTIR spectra of NaCo_x_O_y_, the band at 1639 cm^−1^ was due to the bending mode of the adsorbed water molecules. The bands at 881 and 1442 cm^−1^ were ascribed to the asymmetric stretching Co–OH and Na–O vibrations, respectively. The band at 561 cm^−1^ was due to the Co–O stretching vibrations^36, 40^. The full scan XPS survey of MOFs and derived oxides confirmed the presence of Na in the materials along with C, O, and Co (Fig. 2d). The C peak in NaCo_x_O_y_ materials was due to the adventitious carbon. Na 1s and Na KLL peak intensity increased in the NaCo_x_O_y_ compared to the MOFs due to the loss of carbon after calcination.

The HRXPS spectra of CoBDC and CoBTC are shown in Fig. S4, and the curve-fitting parameters are listed in Tables S3–S5. The HRXPS C 1s spectrum of CoBDC has five contributions at 284.7, 285.8, 287.0, 288.6, and 290.6 eV for C=C/C–H, C–O, –COOCo, –COONa, and π-π* satellite, respectively (Fig. S4a)^41^. The HRXPS Co 2p spectrum of CoBDC has peaks at 782 and 798 eV for Co 2p_3/2_ and Co 2p_1/2_, respectively (Fig. S4b). The HRXPS Co 2p_3/2_ spectrum deconvoluted into two contributions at 781.1 and 782.7 eV for Co^3+^ (49.1%) and Co^2+^ (50.9%), respectively, with two satellites at 785.0 and 788.2 eV^24^. The HRXPS O 1s spectrum of CoBDC has four contributions at 531.5, 532.5, 533.7, and 535.4 eV for O–Co/O–Na, O–C=O, O–H, and H_2_O, respectively (Fig. S4c)^42^. For CoBTC, the HRXPS C 1s spectrum deconvoluted into four peaks at 284.7, 285.9, 288.3, and 290.0 eV for C=C/C–H, C–O, –COOCo, and –COONa, respectively (Fig. S4d). In the HRXPS Co 2p spectrum of CoBTC, the Co^3+^ and Co^2+^ peaks were observed at 781.0 and 782.5 eV with 48.7 and 51.3% contribution, respectively (Fig. S4e). The HRXPS O 1s spectrum of CoBTC has peaks at 531.5, 532.5, and 533.8 eV for O–Co/O–Na, O–C=O, and O–H, respectively (Fig. S4f).

**Figure 3:**
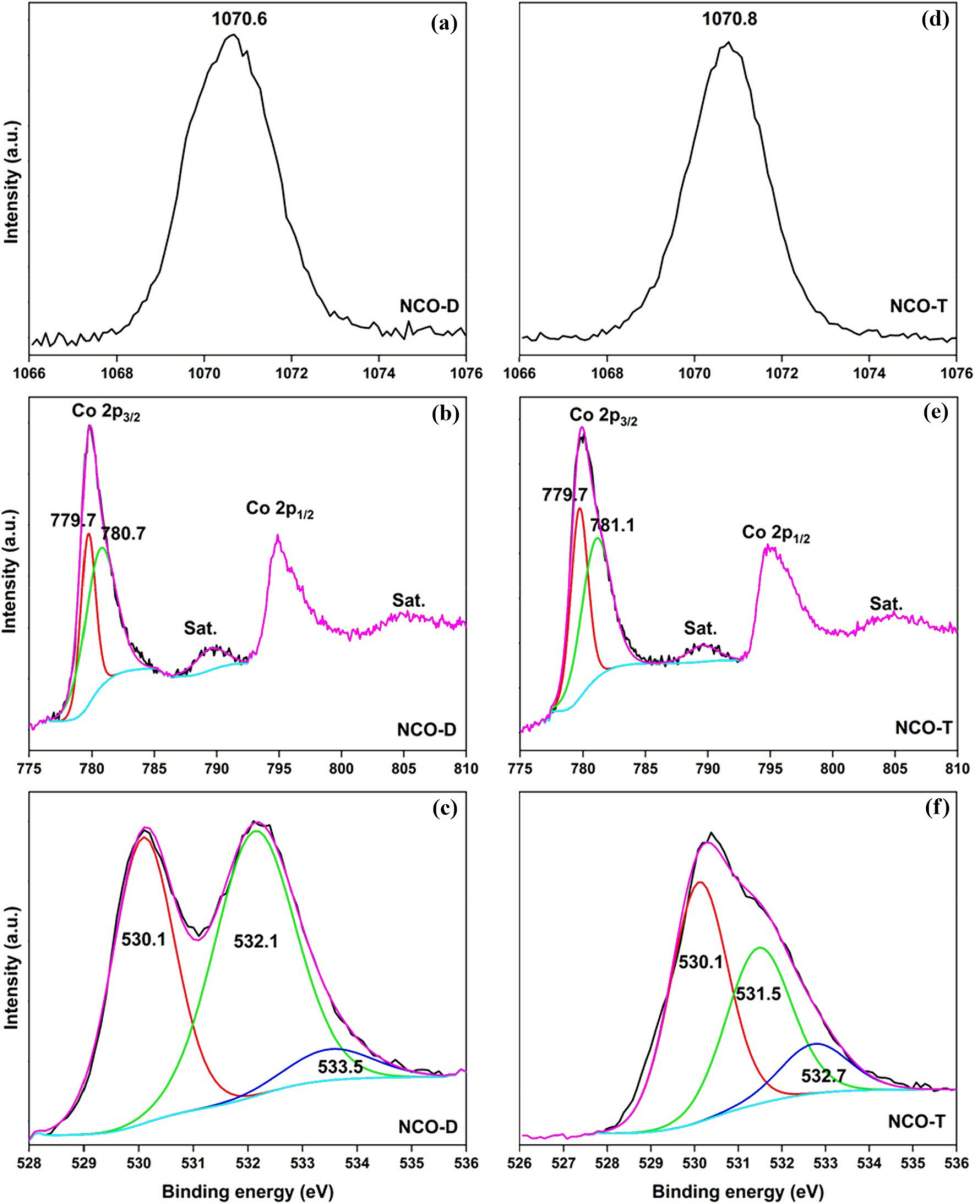
HRXPS (**a**) Na 1s; (**b**) Co 2p; (**c**) O 1s spectra of NCO-D; (**d**) Na 1s; (**e**) Co 2p; (**f**) O 1s spectra of NCO-T.

The HRXPS spectra of NCO-D and NCO-T are shown in Fig. 3, and the curve-fitting parameters are listed in Tables S6 and S7. The HRXPS Na 1s spectra of NCO-D (Fig. 3a) and NCO-T (Fig. 3d) has a peak at 1070.6 and 1070.8 eV, respectively, for Na^+^ ions^43^. The HRXPS Co 2p spectrum of NCO-D has peaks at 779.8 and 794.9 eV with satellite peaks at 789.6 and 804.8 eV for Co 2p_3/2_ and Co 2p_1/2_, respectively (Fig. 3b). The Co 2p_3/2_ peak was deconvoluted into two contributions at 779.7 and 780.7 eV for Co^3+^ (36.4%) and Co^2+^ (63.6%) ions, respectively^44^. The HRXPS O 1s spectrum of NCO-D has three contributions at 530.1, 532.1, and 533.5 eV for O–Co/O–Na, O–H, and H_2_O, respectively (Fig. 3c)^45^. The HRXPS Co 2p spectrum of NCO-T has peaks at 779.7 and 781.1 eV for Co^3+^ (44.1%) and Co^2+^ (55.9%), respectively (Fig. 3e). The HRXPS O 1s spectrum of NCO-T has similar contributions as observed in NCO-D (Fig. 3f). Based on the analysis, NCO-D and NCO-T were NaCo^III^_0.25_Co^II^_0.45_O_1.33_ and NaCo^III^_0.48_Co^II^_0.62_O_1.67_, respectively.

### H_2_S breakthrough studies

**Figure 4:**
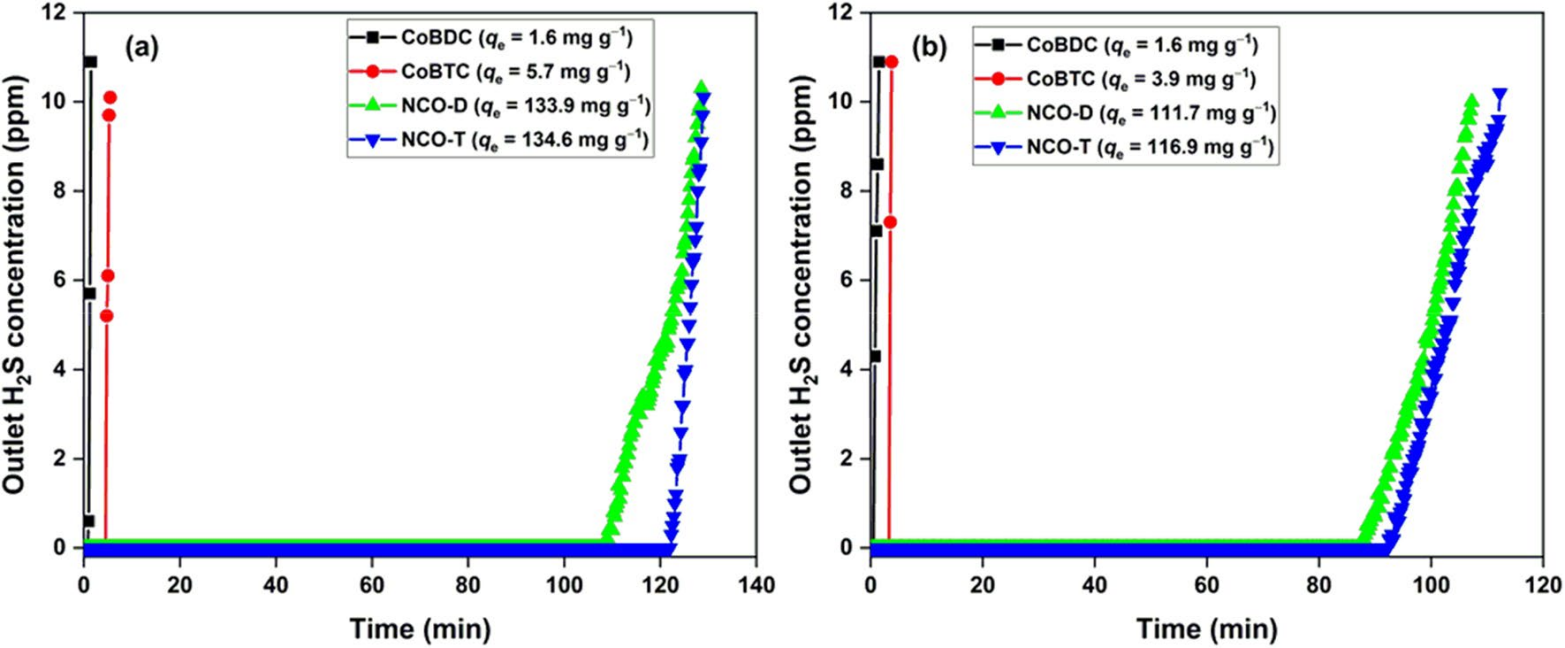
H_2_S breakthrough curves of MOFs and derived metal oxides in (**a**) dry; (**b**) moist conditions. [Adsorbent] = 0.2 g, flowrate = 0.3 L min^−1^.

The breakthrough curves for MOFs and oxides in dry and moist conditions are shown in Fig. 4. CoBDC and CoBTC showed a low adsorption capacity of 1.6 and 5.7 mg g^−1^, respectively, in dry condition (Fig. 4a). The studies dealing with Co-based MOFs for H_2_S removal are absent in the literature. The closest study is the role of Co ions in UiO-67(bipy) for H_2_S removal. The post-synthetic inclusion of Co in the MOF could not substantially improve its H_2_S removal capacity. The Co-sites in a highly porous MOF with a surface area of ~ 2500 m^2^ g^−1^ failed to interact with H_2_S gas^46^. The low adsorption capacity of Co-MOFs in the present study was probably due to the low Co-density and poor accessibility of Co-sites in MOFs for H_2_S interaction. The NCO-D and NCO-T had an adsorption capacity of 133.9 and 134.6 mg g^−1^, respectively, which was a significant improvement compared to MOF precursors. In the moist condition, the adsorption capacity of MOFs and derived oxides decreased. In general, the presence of moisture plays a positive role in the H_2_S adsorption process by dissociating H_2_S molecules in the water film^37, 47^. The adsorption capacity could decrease due to the competitive behaviour of water molecules for the adsorption sites^48^. Moreover, the formation of sulfuric acid could lower the structural integrity of the adsorbent and decrease its adsorption capacity. In the present case, moisture alone can destroy the adsorbent structure due to the hygroscopic nature of sodiated transition metal oxides^49^. Nevertheless, the H_2_S adsorption capacity was satisfactorily preserved even in the presence of moisture. The higher adsorption capacity of NCO-T was due to its comparatively higher surface area than NCO-D. The surface area of nonporous adsorbent is highly relevant in the adsorption of gases. Zheng et al. reported an increased CO_2_ adsorption capacity (1.02–2.83 cm^3^ g^−1^) in KNbWO_6_·H_2_O pyrochlore with an increase in the surface area (1.82–2.90 m^2^ g^−1^) after Sn^2+^ substitution^50^. Thus, the surface area plays a decisive role in the gas adsorption capacity of nonporous materials. The N_2_ adsorption–desorption isotherms of spent NCO-D and NCO-T are available in Fig. S5. The surface area of spent NCO-D and NCO-T was 0.94 and 1.16 m^2^ g^−1^, respectively. The deposition of elemental sulfur and sulfate species was responsible for the decreased surface area. The decreased surface area after H_2_S exposure further supported the fact that a low surface area restricts the gas diffusion process and limits the adsorption capacity.

**Figure 5:**
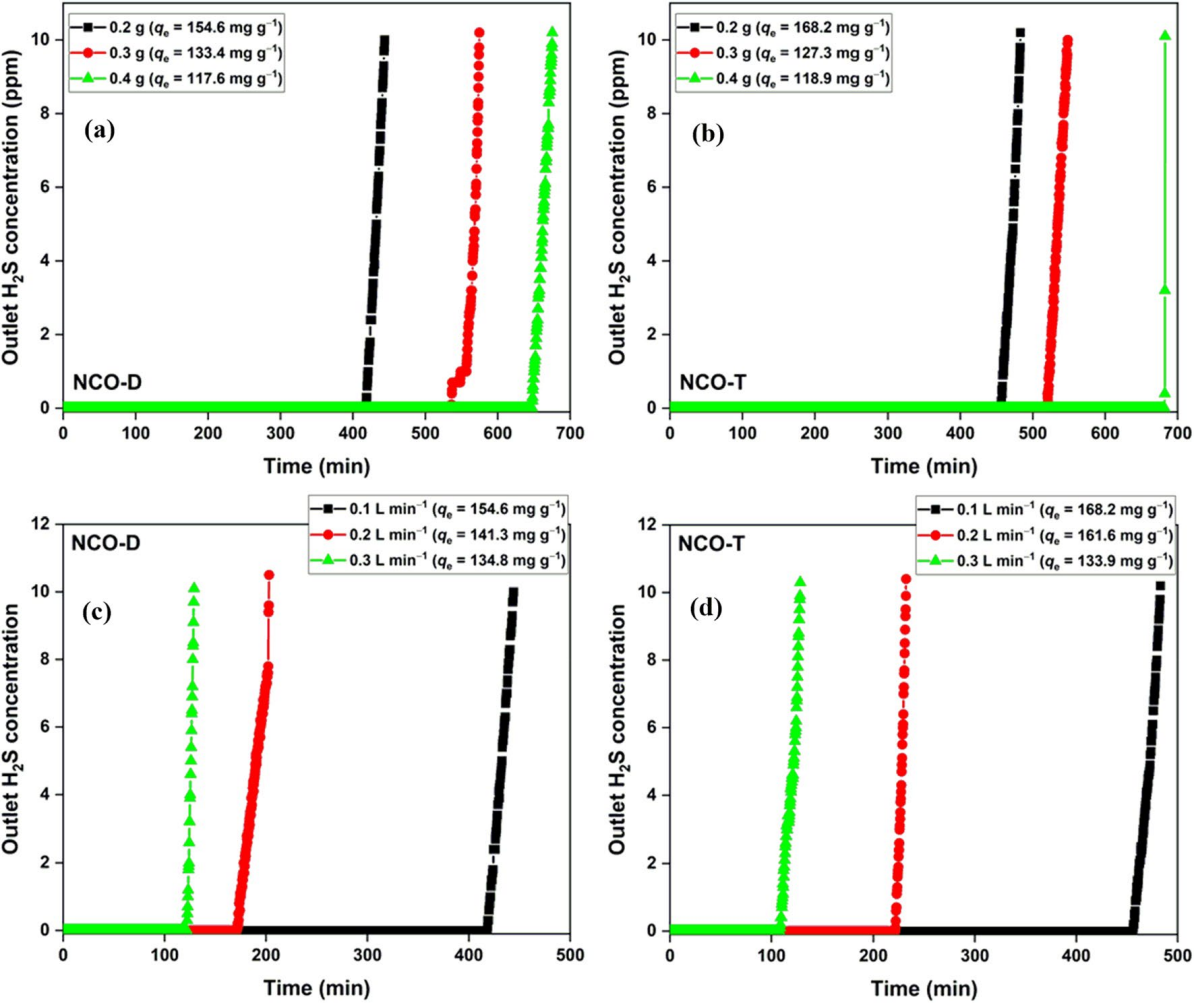
Effect of dosage on the adsorption capacity of (**a**) NCO-D; (**b**) NCO-T, flowrate = 0.1 L min^−1^; effect of flowrate on the adsorption capacity of (**c**) NCO-D; (**d**) NCO-T, [Adsorbent] = 0.2 g.

The effect of dosage and flowrate on the adsorption capacity was studied for NCO-D and NCO-T (Fig. 5). The adsorption capacity was negatively impacted by the increasing adsorbent mass (Fig. 5a,b). The adsorption capacity of 154.6 and 168.2 mg g^−1^ with a 0.2 g dosage reached 117.6 and 118.9 mg g^−1^ with 0.4 g dosage for NCO-D and NCO-T, respectively. The decreased adsorption capacity was due to the formation of dead zones in the bed with the increasing bed loading, which remained unused during the initial phase of the adsorption process^51^. The adsorption capacity decreased with the increasing H_2_S flow rate (Fig. 5c,d). The adsorption capacity of 154.6 and 168.2 mg g^−1^ (0.1 L min^−1^) for NCO-D and NCO-T dropped to 134.8 and 133.9 mg g^−1^ (0.3 L min^−1^), respectively. The decrease in the adsorption capacity with the increasing flow rate was due to the insufficient contact time for adsorbate-adsorbent interactions at a higher flow rate. The impact was stronger due to the low surface area and porosity of the metal oxide adsorbents^52^.

The adsorption capacity of synthesized oxides was compared with the Co-based adsorbents reported in the literature (Table 1). The H_2_S uptake capacity of NCO-D and NCO-T were superior to many of the reported adsorbents. The higher adsorption capacity of the Co_3_O_4_–SiO_2_ composite was due to its high porosity. Only Zn-Co hydroxide had a higher capacity than NCO adsorbents. Nevertheless, the MOF-derived NaCo_x_O_y_ adsorbents reported in these studies are unique and highly effective in removing H_2_S from effluent gases at room temperature.

**Table 1:** The adsorption capacity of cobalt-based adsorbents at room temperature.

Adsorbent	Experimental conditions				*q*_e_ (mg g^−1^)
	*C*_0_ (ppm)	Mass (g)	Flow rate (mL min^−1^)	BTP (%)	
Mesoporous Co_3_O_4_^14^	10,000	0.05	50	0.5	143.0^d^
Co_3_O_4_	10	0.025	#	–	6.0^d^
Zn_3.5_CoO_4.9_					134.0^d^
Zn_2.6_Co_1.5_AlO_6.1_^13^					107.0^d^
Co_3_O_4_–SiO_2_ composite^15^	364	–	100	0.2	189.0^m^
Co_3_O_4_/MCM-41^57^	14,340	0.2	20	100	52.6^d^
Co(OH)_2_	1000	–	500	10	16.2^d^
					26.7^m^
Co(OH)_2_/graphite oxide composite^17^					24.3^d^
					120.3^m^
CoOOH	1000	–	500	10	69.1^d^
					121.8^m^
CoOOH/graphite oxide composite^16^					66.3^d^
					108.2^m^
Zn-Co hydroxide^58^	1000	–	500	10	31.0^d^
					228.1^m^
NCO-D	500	0.2	100	2	154.6^d^
NCO-T [present study]					168.2^d^

^#^Static condition with 20 L of gas equilibrated for 24 h; ^d^Dry condition; ^m^Moist condition.

### Adsorption mechanism

**Figure 6:**
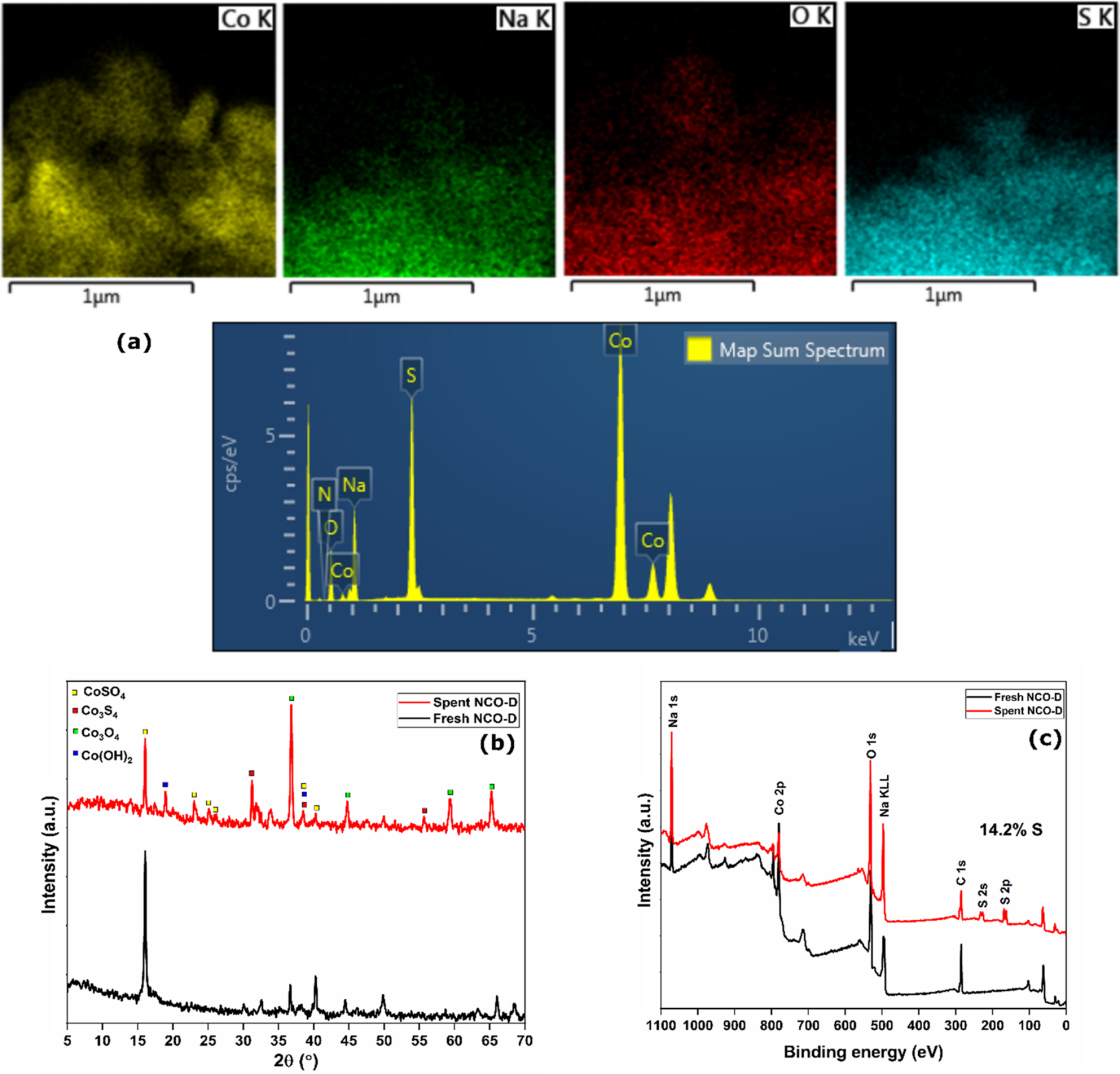
(**a**) TEM-EDS analysis; (**b**) PXRD patterns; (**c**) XPS surveys of spent NCO-D in dry condition.

The distribution of sulfur in the spent NCO-D was probed through TEM-EDS analysis (Fig. 6a). The EDS map has peaks for Co, O, and Na with a new high-intensity peak for S at ~ 2.1 keV. The 2D elemental mapping confirmed the wide distribution of Na, Co, and O in the NCO-D adsorbent. Apart from the constituent elements, uniform distribution of S was observed in the spent adsorbent. The PXRD patterns of fresh and spent NCO-D are shown in Fig. 6b. The major peaks observed at 16.2°, 36.6°, 40.2°, 49.8°, 65.9°, and 68.6° for fresh NCO-D either disappeared or remained with decreased intensity after the H_2_S exposure. Wang et al. have studied the H_2_S oxidation process over Co_3_O_4_. Though the mechanism was probed by XPS analysis, the study suggested at least five products (CoS. CoSO_4_, CoSOH, CoOOH, and elemental sulfur)^15^. Park et al. reported the catalytic oxidation of H_2_S at 350 °C over Co_3_O_4_, where the XRD pattern confirmed the formation of CoSO_4_^53^. Jun et al. reported the formation of Co_9_S_8_ after high-temperature oxidation of H_2_S over ZnCoTiO_4_^54^. Pahalagedara et al. reported the formation of Co_3_S_4_ after the H_2_S desulfurization process over mesoporous Co_3_O_4_ at 200 °C^14^. The PXRD pattern of spent NCO-D has sharp peaks in the entire 15°–70° range. These peaks were assigned to Co_3_S_4_, CoSO_4_, Co_3_O_4_, and Co(OH)_2_ phases. The XPS surveys of fresh and spent NCO-D are shown in Fig. 6c. The XPS survey of spent NCO-D has peaks for S 2p and S 2s at ~ 165 and ~ 234 eV, respectively. The S present in the spent sample accounted for 14.2% of the total atomic composition.

**Figure 7:**
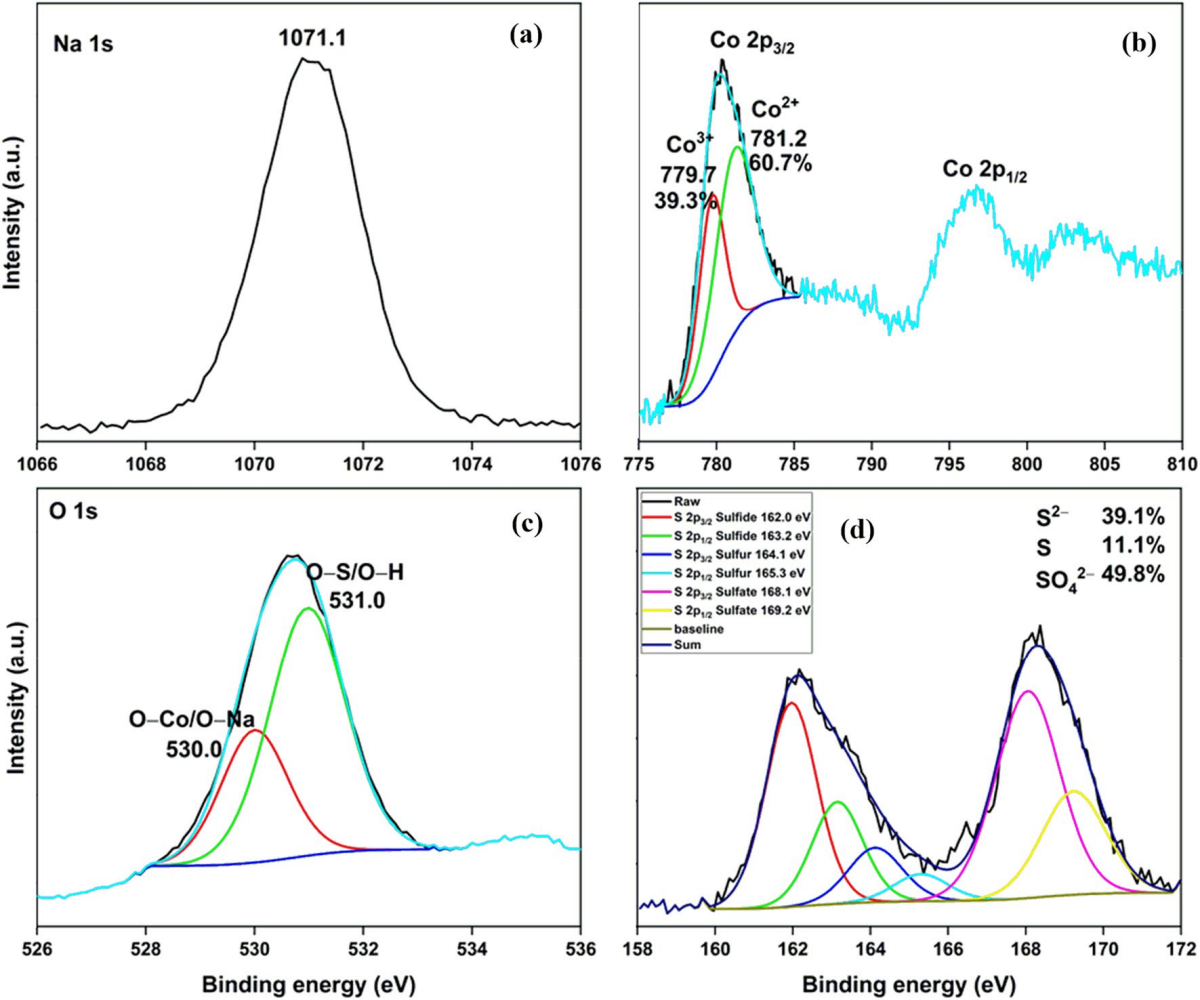
HRXPS (**a**) Na 1s; (**b**) Co 2p; (**c**) O 1s; (**d**) S 2p spectra of spent NCO-D in dry condition.

The HRXPS Na 1s peak at 1070.6 eV for fresh NCO-D shifted to 1071.1 eV for spent NCO-D (Fig. 7a). The shift in the binding energy of Na 1s peak by 0.3 eV was probably due to the redistribution of electron density after the formation of different sulfide and oxide phases of cobalt. In the HRXPS Co 2p spectrum of spent NCO-D, the peaks at 780.3 and 796.7 eV were assigned to the Co 2p_3/2_ and Co 2p_1/2_, respectively (Fig. 7b). The HRXPS Co 2p_3/2_ peak has two contributions at 779.7 and 781.2 eV for Co^3+^ (39.9%) and Co^2+^ (60.7%) sites, respectively. In the fresh NCO-D, the Co^3+^ and Co^2+^ contribution was 36.4 and 63.6%, respectively. The variation in the contributions of Co^3+^ and Co^2+^ sites in the NCO-D was due to the involvement of the Co^3+^/Co^2+^ catalytic cycle in the H_2_S oxidation process. The HRXPS O 1s spectrum of spent NCO-D has two contributions at 530.0 and 531.0 eV for O–Na/O–Co and O–S/O–H, respectively (Fig. 7c). The increase in the hydroxyl density over the adsorbent was most likely due to the interaction of H_2_S molecules with the surface lattice O^2−^ to yield HS^–^ and –OH groups^55^. Moreover, the absence of surface H_2_O in the spent sample hinted towards its full utilization to form sulfates and Co(OH)_2_. The HRXPS S 2p spectra of spent NCO-D is shown in Fig. 7d. The spectrum was fitted into six peaks for different sulfur species. The peaks at 162.0 and 163.2 eV were assigned to the S 2p_3/2_ and S 2p_1/2_ of sulfide species, respectively, which were bound to Co ions in Co_3_S_4_^15, 56^. The sulfide species was primarily formed due to the reactive interaction of adsorbed H_2_S molecules with the lattice oxygen. The peaks at 164.1 and 165.3 eV were attributed to the S 2p_3/2_ and S 2p_1/2_ of elemental sulfur, respectively^18^. The peaks at 168.1 and 169.2 eV were assigned to the S 2p_3/2_ and S 2p_1/2_ of sulfate ions, respectively. These sulfate ions were considered as CoSO_4_, observed in the PXRD pattern as well^18, 37^. Thus, all three major sulfur species were conclusively detected in the XPS analysis. Moreover, the sulfide, sulfur, and sulfate contribution in the total sulfur content were 39.1, 11.1, and 49.8%, respectively. Based on the above discussion, the following reactions have been proposed for the H_2_S removal over NCO-D.

2 $$lattice\;(O^{2 - } ) + H_{2} S \to - OH + HS^{ - }$$

3 $$2Co^{3 + } + S^{2 - } \to 2Co^{2 + } + S$$

4 $$2Co^{2 + } + \frac{1}{2}O_{2} \to 2Co^{3 + } + O^{2 - }$$

5 $$S + \frac{1}{2}O_{2} + H_{2} O \to H_{2} SO_{4}$$

6 $$NaCo_{x} O_{y} + H_{2} O \to NaOH + Co_{3} O_{4} + CoO$$

7 $$Co_{3} O_{4} + 4H_{2} O \to 3Co(OH)_{2}$$

8 $$CoO + H_{2} SO_{4} \to CoSO_{4} + H_{2} O$$

## Conclusion

We have reported a novel approach for the fabrication of NaCo_x_O_y_ adsorbents by air calcination of (Na,Co)-organic frameworks. NaCo_x_O_y_ were formed irrespective of the type of organic linkers used in the MOF precursor. Moreover, the oxides crystallized as microsheets of 100–200 nm thickness with the presence of some polyhedral nanocrystals. These macroporous oxides have a surface area in the range of 1.15–1.90 m^2^ g^−1^. X-ray photoelectron spectroscopy (XPS) analysis confirmed the near equal presence of Co^2+^ and Co^3+^ sites in MOFs, which were largely preserved in the NaCo_x_O_y_. The maximum adsorption capacity of 168.2 mg g^−1^ was recorded for NCO-T in dry conditions. The competitive nature of water molecules led to the decrease in adsorption capacity in moist condition. The adsorption capacity decreased with the increasing flow rate and bed loading due to the insufficient contact time for adsorbate-adsorbent interactions and the formation of dead zones, respectively. TEM-EDAX analysis confirmed abundant and uniform distribution of sulfur in the adsorbent. PXRD analysis of the spent sample suggested the formation of Co_3_S_4_, CoSO_4_, Co_3_O_4_, and Co(OH)_2_ after the H_2_S exposure. The products of the H_2_S adsorption-oxidation process were further confirmed by XPS analysis. Thus, we have reported highly efficient adsorbents for the adsorptive-oxidative removal of H_2_S gas in ambient conditions.

## Supplementary Information

Supplementary Information.
