# Supplementary material for: Metal organic framework derived NaCoxOy for room temperature hydrogen sulfide removal
Source: Sci Rep. 2021 Jul 19;11:14740. doi: 10.1038/s41598-021-94265-7 (PMC8290053; doi:10.1038/s41598-021-94265-7)
Supplement: Supplementary file 1 — Supplementary Information. [file 41598_2021_94265_MOESM1_ESM.docx]

**Metal organic framework derived NaCo_x_O_y_ for room temperature hydrogen sulfide removal**

Nishesh Kumar Gupta^a,b^, Jiyeol Bae^a,b*^, Kwang Soo Kim^a,b^

1. *University of Science and Technology (UST), Daejeon, Republic of Korea*
2. *Department of Land, Water, and Environment Research, Korea Institute of Civil Engineering and Building Technology (KICT), Goyang, Republic of Korea*


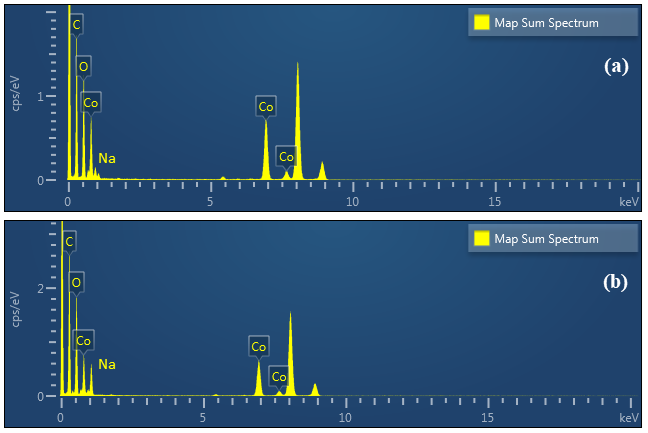


**Fig. S1.** TEM-EDS analysis of (**a**) CoBDC; (**b**) CoBTC.


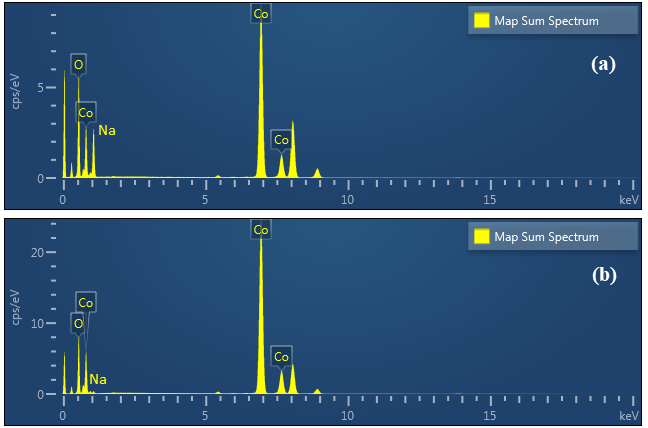


**Fig. S2.** TEM-EDS analysis of (**a**) NCO-D; (**b**) NCO-T.





**Fig. S3.** PXRD patterns of Na_2_O, Co_3_O_4_, and NaCo_2_O_4_.


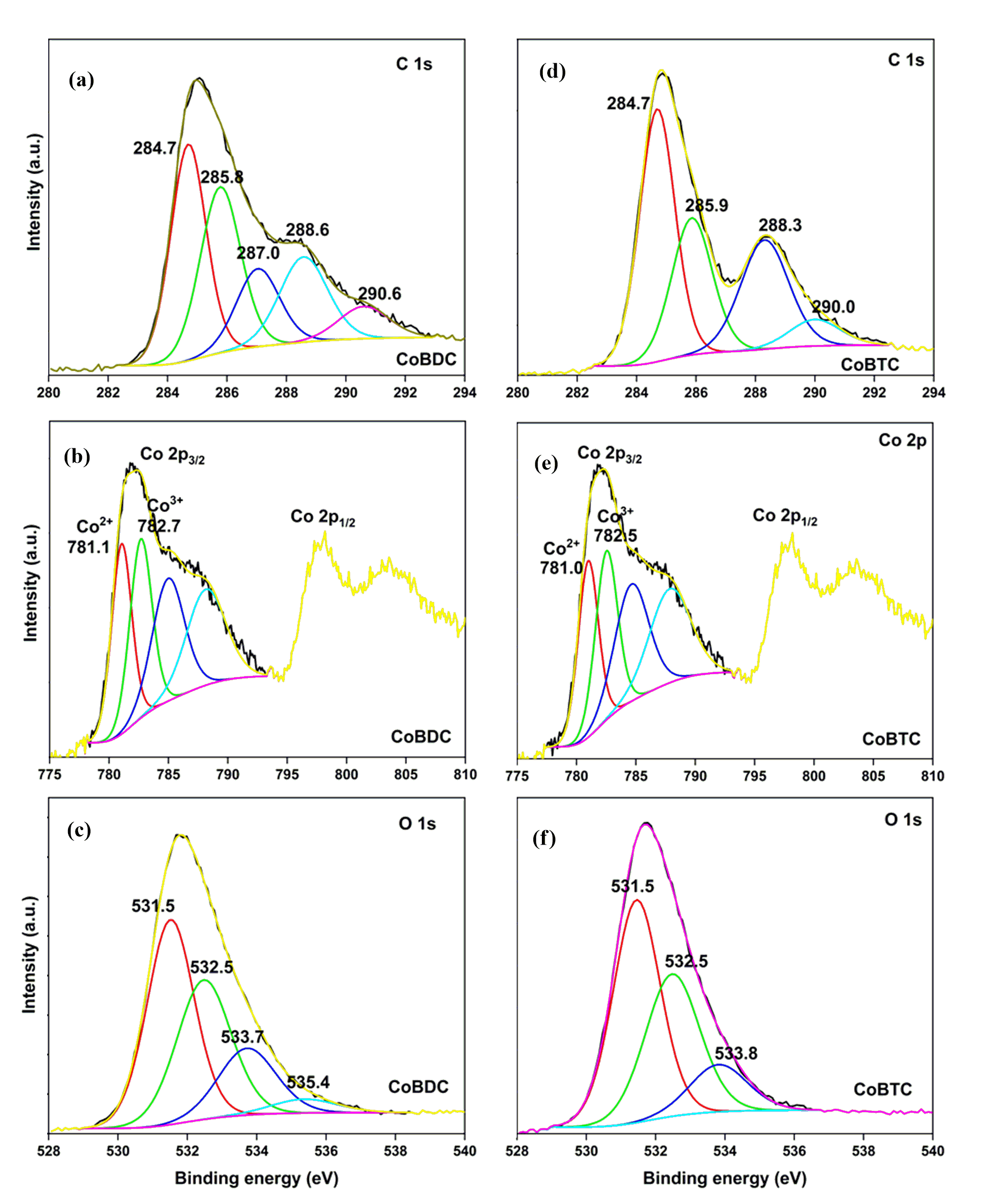


**Fig. S4.** HRXPS (**a**) C 1s; (**b**) Co 2p; (**c**) O 1s spectra of CoBDC; (**d**) C 1s; (**e**) Co 2p; (**f**) O 1s spectra of CoBTC.





**Fig. S5.** N_2_ adsorption-desorption isotherms of spent NCO-D and NCO-T.

**S. Table 1.** Elemental composition of MOF-derived oxides.

| **Adsorbent** | **Elements (At. %)** | | |
| --- | --- | --- | --- |
|  | **O 1s** | **Co 3p** | **Na 1s** |
| **NCO-D** | 58.57 | 16.98 | 24.29 |
| **NCO-T** | 61.44 | 20.09 | 18.48 |

**S. Table 2.** Surface area and porosity of MOFs and derived oxides.

| **Adsorbent** | **Surface area (m^2^ g**^–^**^1^)** | **Pore volume (cm^3^ g**^–^**^1^)** | **Pore diameter (nm)** |
| --- | --- | --- | --- |
| **CoBDC** | 18.26 | 0.106 | 23.1 |
| **CoBTC** | 6.85 | 0.032 | 19.0 |
| **NCO-D** | 1.15 | 0.004 | 13.1 |
| **NCO-T** | 1.90 | 0.008 | 17.9 |

**S. Table 3.** Curve fitting parameters from C 1s spectra of Co-MOFs

| **Samples** | **Assignment** | **E_B_ (eV)** | **FWHM (eV)** | **At. %** |
| --- | --- | --- | --- | --- |
| **CoBDC** | **C1s** _C=C,C–H_ | 284.7 | 1.4 | 32.6 |
|  | **C1s** _C–O_ | 285.8 | 1.6 | 28.6 |
|  | **C1s** _–COOCo_ | 287.0 | 1.7 | 14.4 |
|  | **C1s** _–COONa_ | 288.6 | 1.9 | 17.5 |
|  | **C1s** _π-π*_ | 290.6 | 2.0 | 6.9 |
| **CoBTC** | **C1s** _C=C,C–H_ | 284.7 | 1.4 | 42.5 |
|  | **C1s** _C–O_ | 285.9 | 1.6 | 26.3 |
|  | **C1s** _–COOCo_ | 288.3 | 1.9 | 24.9 |
|  | **C1s** _–COONa_ | 290.0 | 2.0 | 6.4 |

**S. Table 4.** Curve fitting parameters from Co 2p spectra of Co-MOFs

| **Samples** | **Assignment** | **E_B_ (eV)** | **FWHM (eV)** | **At. %** |
| --- | --- | --- | --- | --- |
| **CoBDC** | **Co2p_3/2_** _Co3+_ | 781.1 | 2.0 | 49.1 |
|  | **Co2p_3/2_** _Co2+_ | 782.7 | 2.2 | 50.9 |
|  | **Co2p_3/2_** _Satellite_ | 785.0 | 3.2 | - |
|  | **Co2p_3/2_** _Satellite_ | 788.2 | 3.9 | - |
| **CoBTC** | **Co2p_3/2_** _Co3+_ | 781.0 | 2.0 | 48.7 |
|  | **Co2p_3/2_** _Co2+_ | 782.5 | 2.2 | 51.3 |
|  | **Co2p_3/2_** _Satellite_ | 784.6 | 3.4 | - |
|  | **Co2p_3/2_** _Satellite_ | 787.9 | 4.1 | - |

**S. Table 5.** Curve fitting parameters from O 1s spectra of Co-MOFs

| **Samples** | **Assignment** | **E_B_ (eV)** | **FWHM (eV)** | **At. %** |
| --- | --- | --- | --- | --- |
| **CoBDC** | **O1s** _O–Co/O–Na_ | 531.5 | 1.6 | 44.0 |
|  | **O1s** _O–C=O_ | 532.5 | 1.8 | 34.4 |
|  | **O1s** _O–H_ | 533.7 | 1.9 | 17.5 |
|  | **O1s** _H2O_ | 535.4 | 2.2 | 4.1 |
| **CoBTC** | **O1s** _O–Co/O–Na_ | 531.5 | 1.6 | 50.1 |
|  | **O1s** _O–C=O_ | 532.5 | 1.8 | 37.0 |
|  | **O1s** _O–H_ | 533.8 | 1.9 | 12.9 |

**S. Table 6.** Curve fitting parameters from Co 2p spectra of NCO.

| **Samples** | **Assignment** | **E_B_ (eV)** | **FWHM (eV)** | **At. %** |
| --- | --- | --- | --- | --- |
| **NCO-D** | **Co2p_3/2_** _Co3+_ | 779.7 | 1.4 | 36.4 |
|  | **Co2p_3/2_** _Co2+_ | 780.7 | 2.9 | 63.6 |
|  | **Co2p_3/2_** _Satellite_ | 789.1 | 3.0 | - |
| **NCO-T** | **Co2p_3/2_** _Co3+_ | 779.7 | 1.6 | 44.1 |
|  | **Co2p_3/2_** _Co2+_ | 781.1 | 2.8 | 55.9 |
|  | **Co2p_3/2_** _Satellite_ | 789.6 | 3.0 | - |

**S. Table 7.** Curve fitting parameters from O 1s spectra of NCO.

| **Samples** | **Assignment** | **E_B_ (eV)** | **FWHM (eV)** | **At. %** |
| --- | --- | --- | --- | --- |
| **NCO-D** | **O1s** _O–Co/O–Na_ | 530.1 | 1.3 | 42.6 |
|  | **O1s** _O–H_ | 532.1 | 1.7 | 51.1 |
|  | **O1s** _H2O_ | 533.5 | 1.8 | 6.3 |
| **NCO-T** | **O1s** _O–Co/O–Na_ | 530.1 | 1.5 | 50.5 |
|  | **O1s** _O–H_ | 531.5 | 1.7 | 37.0 |
|  | **O1s** _H2O_ | 532.7 | 1.8 | 12.5 |
